# HumiR: Web Services, Tools and Databases for Exploring Human microRNA Data

**DOI:** 10.3390/biom10111576

**Published:** 2020-11-20

**Authors:** Jeffrey Solomon, Fabian Kern, Tobias Fehlmann, Eckart Meese, Andreas Keller

**Affiliations:** 1 Chair for Clinical Bioinformatics, Saarland University, 66123 Saarbrücken, Germany; s8jesolo@stud.uni-saarland.de (J.S.); fabian.kern@ccb.uni-saarland.de (F.K.); tobias.fehlmann@ccb.uni-saarland.de (T.F.); 2 Institute for Human Genetics, Saarland University, 66421 Homburg, Germany; Eckart.Meese@uks.eu; 3 Center for Bioinformatics, Saarland University, 66123 Saarbrücken, Germany; 4 Department of Neurobiology, Stanford University, Palo Alto, CA 94305, USA

**Keywords:** microRNAs, bioinformatics, web servers, non-coding RNA, bioinformatics

## Abstract

For many research aspects on small non-coding RNAs, especially microRNAs, computational tools and databases are developed. This includes quantification of miRNAs, piRNAs, tRNAs and tRNA fragments, circRNAs and others. Furthermore, the prediction of new miRNAs, isomiRs, arm switch events, target and target pathway prediction and miRNA pathway enrichment are common tasks. Additionally, databases and resources containing expression profiles, e.g., from different tissues, organs or cell types, are generated. This information in turn leads to improved miRNA repositories. While most of the respective tools are implemented in a species-independent manner, we focused on tools for human small non-coding RNAs. This includes four aspects: (1) miRNA analysis tools (2) databases on miRNAs and variations thereof (3) databases on expression profiles (4) miRNA helper tools facilitating frequent tasks such as naming conversion or reporter assay design. Although dependencies between the tools exist and several tools are jointly used in studies, the interoperability is limited. We present HumiR, a joint web presence for our tools. HumiR facilitates an entry in the world of miRNA research, supports the selection of the right tool for a research task and represents the very first step towards a fully integrated knowledge-base for human small non-coding RNA research. We demonstrate the utility of HumiR by performing a very comprehensive analysis of Alzheimer’s miRNAs.

## 1. Introduction

In almost all organisms, bacteria, viruses, plants, animals and mammals, research on small non-coding RNA is performed. Among the different classes of small non-coding RNAs, microRNAs (miRNAs) are among the best studied ones. The current state-of-the-art on miRNA research has been published by Bartel in 2018 [1].

Modern high throughput approaches, most importantly massively parallel sequencing, are applied to generate steadily increasing data sets. A search in the sequence read archive (SRA), one of the most frequent repositories for small RNA sequencing data, yields 95,015 data sets. The following list of organisms is covered by over 1000 samples deposited in SRA: Homo sapiens (46,007 samples); Mus musculus (15,758 samples); Rattus norvegicus (2534 samples); Bos taurus (2307 samples); Arabidopsis thaliana (1683 samples); Sus scrofa (1328 samples);

Drosophila melanogaster (1130 samples); Caenorhabditis elegans (1120 samples). Altogether, 115 TB of sequencing data on small RNAs are deposited in SRA, covering 2 × 10^12^ reads.

The flood of data calls for advanced computational solutions. Chen and co-workers curated 95 review papers covering about 1000 miRNA bioinformatics tools [2]. A great repository that collects an overview of many different miRNA tools from a broad range of applications is Tools4miRs [3]. Although many of the tools are independent of the organism or support several organisms, a certain focus on Homo Sapiens can be recognized. This is motivated by the fact that most and the largest data sets are available for humans as sketched above. The set of tools includes, but is not necessarily limited to, approaches for quantifying miRNAs and other non-coding RNAs, prediction of new representatives from the classes, discovery of isomiRs and other isoforms, prediction of targets and target pathways or the discovery of arm shift and arm switch events. From miRNA research in general, especially, however, from the ever-growing data sets and the new tools, databases on miRNAs are built.

The most prominent and referenced database is the miRBase [4,5] but other databases have also been developed to complement miRBase (MirGeneDB [6] or miRCarta [7] as summarized in [8]). Currently, it is estimated that around 2300 human miRNAs exist that are yet not completely contained in these data bases [9]. In addition to databases with a focus on miRNAs, other resources contain information on predicted or validated targets, variants in miRNAs, isoforms or other structural features. In addition to respective databases, data warehouses have also been generated as resources for miRNA expression patterns, including expression profiles for different organs, tissues, cell types and other specimen types. Here, we do not want to provide anything close to a complete overview of available tools and databases. These can be found in one of the many excellent review articles and tool comparisons on the different topics [2,10,11,12,13,14,15,16,17,18,19].

Over the past decade we have implemented tools and resources that are dedicated to foster the analysis and understanding of miRNAs. The joint paradigm of all solutions is in that they must be available as a web service and not require any installation of client-side software. For these tools, the focus has been on Homo sapiens. A limitation of the tools is their very limited interoperability. For example, one tool might generate an output that can directly be processed by another tool. Frequently, researches might benefit, however, not even being aware that an interesting downstream processing tool or data base with relevant information exists. It is our ambition to strengthen the interoperability and provide better access to the tools and data warehouses. As a first step, we wrapped all web services and databases in docker containers. With the ongoing update of the respective solutions, we start to introduce a growing set of RESTful Application Programming Interface. The final result is anticipated to be a central knowledge base and resource for human miRNA research. Here we introduce HumiR, a web presence that puts the different resources in context, describe the different resources and facilitates the selection of the right tools for users. Notably, we also do not intend to benchmark a study and compare it to competing or complementary tools. This is done within the respective original and updated manuscripts. In contrast, we emphasize that alternative solutions exist that may even have advantages when compared to our tool. A great example is our miRNA pathway enrichment tool miEAA [20] where we explicitly notify users to also test their results with the TAM tool developed in the Cui lab [21]. Of course, we mention selected alternatives to our tools in the respective sections below. The focus of this work is thus more to present the tools in context to each other towards a fully integrated solution.

In the following, we will first group the tools into four main categories: (1) miRNA analysis tools (miRMaster [22], miEAA [20], miRTargetLink, miRSwitch, novoMiRank); (2) databases on miRNAs and variations thereof (miRCarta, miRSNPdb, miRPathDB, miRATbase); (3) databases on expression profiles of miRNAs (TissueAtlas, CellTypeAtlas, ATmiRes); (4) miRNA helper tools facilitating frequent tasks, such as miRNA naming conversion or reporter assay design (miRTaH, miRBaseConverter, miBlast). In each category we will briefly describe the task of the tools and databases. In the second section, we will introduce the HumiR resource and how it can be used by researchers. In the third section, we will then describe dependencies between the tools and exemplify workflows through the different tools. Finally, we will provide an outlook on how we will further improve and automate the interoperability of the tools via APIs towards a fully integrated knowledge base.

## 2. Experiments and Methods

We implemented HumiR, a web presence as entry point to human miRNA research (https://www.ccb.uni-saarland.de/humir/). The web server was implemented using a dockerized version of Django Web Framework (v2.2) running on Python 3.7. The frontend user-interface was built using ReactJS (v16.13) and the Material-UI (v4.9) framework, and bundled by Webpack (v4.25). An Nginx docker container is acting as proxy server in front of the Gunicorn WSGI HTTP server (19.8) providing access to the Django application. The HumiR web interface has been tested with the following browsers: Firefox (version 82.0.3), Safari (version 13.1.1), Google Chrome (Version 87.0.4280.67). The availability of HumiR is monitored on a daily basis by automated API testing.

## 3. Results

### 3.1. The Four Categories of Tools

**Figure 1:**
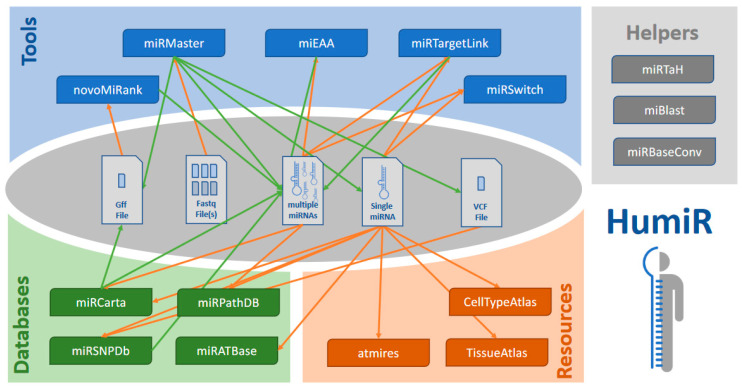
The four classes of tools as well as the respective tools. The middle of the figure presents the different file types, input or output types for the tools. Orange arrows denote a tool input, e.g., NovoMiRank only works with *.gff files. Orange arrows denote an output of a tool. Theoretically, walks through this graph can be generated, following this scheme: a tool gets a file type as input and produces another filetype as output, which in turn is again used as input for the next tool.

The first set of tools take either high throughput data directly (fastq) or sets of miRNAs as input to process them. While many of the tools also support an analysis of single miRNAs, the actual key criterion is the processing of large data sets. The second set of tools relates to databases that contain information on miRNA sequences or variations thereof, target and target pathway databases. The third set are databases that host expression profiles on miRNAs. Finally, the last set of tools are small helper tools that facilitate important tasks quickly. The tools are shown in context to each other in Figure 1 and summarized in Table 1.

### 3.2. miRNA Analysis Tools

**miRMaster** [22,23]: miRMaster is an all-in-one tool for the analysis of raw sequencing data stored in fastq files. The files are compressed client-side in javascript, uploaded and then processed. The tool is tailored for Illumina and BGI/MGISEQ data and also supports samples with unique molecular identifiers as, for example, are implemented in the latest template switch protocols [24]. miRMaster quantifies known miRNAs, miRNAs, tRNAs and other small RNAs, has its own prediction engine for new miRNAs, maps the data to viruses and bacteria, detects isoforms and reports SNVs in miRNAs. Importantly, case control analyses are also supported and differentially expressed features are reported. As of October 2020, over 1400 studies have been performed with miRMaster and almost 100,000 samples (50,000 unique samples) have been processed covering a total of 1.3 trillion reads (600 Billion unique reads). Alternative tools include among others sRNAbench [25], CPSS2 [26] or CBS-mirSeq [27].

**miEAA** [20,28]: miEAA is a miRNA enrichment tool. It works conceptually similar to gene set enrichment analysis originally introduced by Subramanian [29]. The tool expects either a list of miRNAs or a set of miRNAs which is then compared to a reference set. In the first case, the unweighted GSEA is performed with an exact computation of p-values [30]. In the second case the hypergeometric distribution is computed to get a p-value. miEAA hosts over 15,000 different functional categories and outputs graphics and tables detailing the pathway enrichment scores. A comprehensive alternative to miEAA is the TAM tool developed by the Cui lab [21].

**miRTargetLink** [31]: miRTargetLink takes either a single miRNA, a single gene, a set of miRNAs or a set of genes and computes from there the miRNA-target network containing all participants provided by the user. Predicted targets, targets with weak evidence such as microarrays or targets with stronger evidence such as reporter assays can be chosen between. Alternatives to miRTargetLink include miRNet 2.0 [32] or miRViz [33].

**miRSwitch** [34]: miRSwitch is a tool that takes miRNA expression data and from there computes whether miRNAs perform a shift or switch in terms of the dominant arm. It also offers a comprehensive background knowledge base that, e.g., hosts arm switch events between different tissues. The input to miRSwitch is a miRNA expression matrix, but if only the knowledge base is queried a single miRNA may, of course, also be used as input.

**NovoMiRank** [35]: Tools such as miRMaster often deliver long sets of potential novel miRNAs. However, many of these are likely false positives that are introduced, e.g., by library prep bias, low quality samples or computational artifacts [36,37]. To this end, we implemented NovoMiRank. The tool takes new miRNA candidates as a gff file and computes for each miRNA the likelihood of being either a true or false positive.

### 3.3. Qualitative miRNA Data Bases

**miRCarta** [7]: miRCarta is a data base that has the ambition to store not only all current miRNAs, but also all potential candidates in that it is a very sensitive complementation to the very specific and highly curated miRGeneDB [38]. miRCarta stores miRNAs and miRNA candidates, offers an organism independent nomenclature that is unambiguous and bears many additional features such as targets, miRNA families and others. miRCarta can be queried by using any standard miRNA ID, or a gff3 file can be uploaded and processed. Another alternative to miRCarta and miRGeneDB is the reference database miRBase [4,5,39].

**miRSNPdb** [10]: miRSNPdb is a database of predicted miRNA-target interactions, in which we have assessed all SNPs which may occur in mature miRNAs and/or their target regions, and have predicted any target gains or losses due to these SNPs. As input, users can provide a miRNA ID, a gene, or a rs ID representing a SNP. Furthermore, users can also upload VCF files as generated in typical DNA sequencing studies to perform a batch analysis. Alternatives are, e.g., miRSNP [40] or PolymiRTS [41]

**miRPathDB** [42,43,44]: The miRNA target pathway dictionary that has led to the development of miRpathDB is a resource for storing target pathways of miRNAs. In brief, for each miRNA different target sets, strong evidence, weak evidence and predicted targets are pre-computed by GeneTrail [45]. Currently, the database hosts dozens of millions of respective miRNA to target pathway associations. Users can query the database by entering a miRNA identifier. Moreover, sets of miRNAs can be provided and heat maps are generated that show which pathways are targeted by those miRNAs. As a final feature, a maximal coverage analysis via integer linear programming has been introduced. Users determine a maximum number of miRNAs and provide a gene set and the tool computes the minimal number of miRNAs targeting the maximal number of genes provided.

**miRATbase:** miRATbase is our approach to complement the purely in silico database miRPathDB with fully validated experimental target pathways of single miRNAs. The idea is that each gene targeted by a miRNA and on the respective pathway is validated by a reporter assay approach, in that miRATbase also contains a comprehensive database with results validating miRNA gene interactions by luciferase assays. Of course, there exist substantially larger databases miRTarBase, for example, [46] contains a factor more of miRNA gene interactions. There are two differences however. The first one is that for single miRNAs, we provide a very large numbers of targets that are generated in a highly standardized manner. The average of miRNA gene interactions per manuscript contained in the miRTarBase is only 1.6. The second point is the availability of negative interactions. While only 3% of all reported miRTarBase reporter assays are negative, we include all negative validation experiments. This makes the data sets of high value for researchers that want to validate their tools.

### 3.4. Quantitative miRNA Data Bases

**TissueAtlas** [47]: The miRNA tissue atlas is a repository that hosts expression profiles of miRNAs across more than 30 organs and tissues. A unique point is that the organs have largely been collected from the same bodies, excluding inter-individual variations. Researchers can query the atlas by entering any miRNA identifier and get back different expression statistics that can be interactively adjusted. While the TissueAtlas has become our most cited miRNA resource, the current version is still limited to microarray data. The next release will rely on more tissues, more bodies and a much deeper insight in that we use next-generation sequencing as the experimental technique.

**CellTypeAtlas** [48,49]: Similar to the TissueAtlas we also generated a resource for miRNA expression in different blood cell types. Again, researchers can query the resource by entering any miRNA identifier. In addition to the different cell types, an important factor is the influence of the purification techniques. MiRNA profiles also vary depending on whether positive selection, negative selection or FACS sorting has been used.

**ATmiRes** [50]: We also developed a resource that investigates miRNA expression patterns in ancient specimens. The most relevant being, of course, miRNA patterns in solid tissue biopsies of the Tyrolean Iceman. While these patterns that still indicate a certain organ specificity are of limited value for actual research questions, they nonetheless demonstrate that selected miRNAs are highly conserved.

### 3.5. miRNA Helper Tools

**miRTaH:** In our research, we recognized that one time-consuming task is to design constructs for reporter assays. An analysis of the miRTarBase shows that around 6000 manuscripts report, on average, 1.6 validated target genes by reporter assays. We thus anticipated a need of others for automated reporter assay design. As a result, we developed the miRNA Target Assay Helper miRTaH.

**miRBaseConverter:** Another time intensive task is to map identifiers between different miRBase versions. We thus developed a tool for the batch conversion. A list of miRNA ids is provided as input, original and desired output version are given and the mapped ids are returned. This is, e.g., of immense help if an experiment with a microarray using V20 of the miRBase has been carried out and a certain tool requires version 18 as input.

**miBlast:** Finally, another task is to understand whether a small piece of RNA, e.g., predicted from small RNA sequencing results, has already been reported as a miRNA. We thus developed miBlast. Here, you can enter a sequence or a set of sequences and as output you get the respective hits from miRCarta.

### 3.6. The HumiR Web Presence

**Figure 2:**
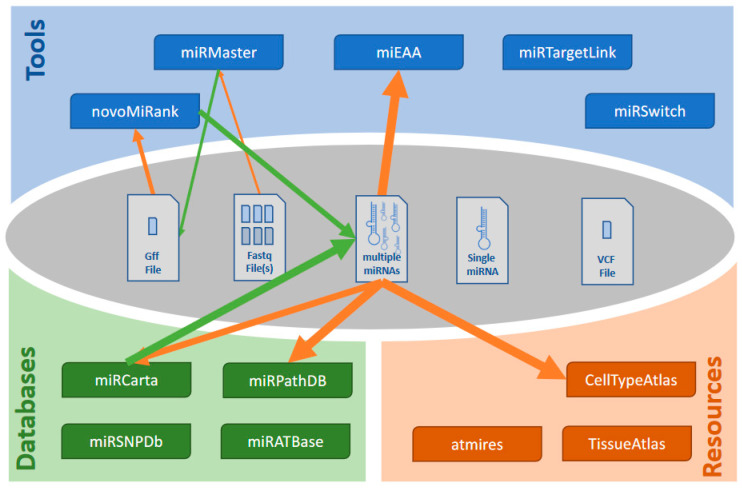
One walk and workflow. Taking the setup from Figure 1, an example workflow is presented. The thickness of the arrow represents the steps in the workflow. Starting from a fastq file miRMaster is used to predict new miRNAs. From these new miRNAs a *.gff file is extracted and used to score the most likely true positive miRNAs. Those most likely true positive miRNAs can be checked for consistency with miRCarta and the consistent miRNAs from miRCarta can then be checked with respect to their expression profiles, or target pathways can be evaluated. Pathway enrichment using miEAA is also evaluated.

As sketched in the introduction our long-term vision is an integrated knowledge base and analysis workflow for human miRNAs. We detail our vision in the fourth section. As a very first step towards this challenging goal, we developed the HumiR web presence available at https://www.ccb.uni-saarland.de/humir/. Here, we collect the tools that we mention in the present work in the respective four categories. It is thought to provide an overview on the available tools for researchers and should serve as an entry point in database queries or for running analysis tasks. To facilitate the selection of the available tools for a given data type we provide all potential input types on top of the HumiR web presence. These include single miRNAs, sets of miRNAs, Lists of miRNAs, GFF files, VCF files and fastq files. The user selects his data type by clicking the icon and all tools that support this input type are highlighted while all other tools are disabled. If single miRNAs are provided as the input type, all data bases and tools besides miEAA, miRMaster and NovoMiRank are available. For fastq, only miRMaster is available, since currently miRMaster is the only tool that works on fastq files directly. In addition, the web presence contains descriptions of the tools and other useful information. The web interface of HumiR is presented in Figure 2.

**Table 1:** Tool overview.

Category	Tool	Reference	Organism	ncRNA Class	Brief Description/Link
**Analysis**	miRMaster	[22,23]	human	miRNA, piRNA, tRNA (+5 others)	non-coding RNA analysis from fastq sequencing files https://www.ccb.uni-saarland.de/mirmaster
	miEAA	[20,28]	human, mouse, rat (+7 others)	miRNA	miRNA pathway analysis https://www.ccb.uni-saarland.de/mieaa
	miRTarget Link	[31]	human	miRNA	analysis of miRNAs and genes in networks https://www.ccb.uni-saarland.de/mirtargetlink
	miRSwitch	[34]	human	miRNA	analysis of arm switch and shift events https://www.ccb.uni-saarland.de/mirswitch
	NovoMiRank	[35]	human	miRNA	ranking of novel miRNA candidates https://www.ccb.uni-saarland.de/novomirank
**Database**	miRCarta	[7]	all from miRBase	miRNA	comprehensive collection of miRNAs and miRNA candidates https://www.ccb.uni-saarland.de/mircarta
	miRSNPdb	[10]	human	miRNA	mutations in human miRNAs https://www.ccb.uni-saarland.de/mirsnp
	miRPathDB	[42,43,44]	human, mouse	miRNA	target pathways and categories of miRNAs https://mpd.bioinf.uni-sb.de
	miRATbase	NA	human	miRNA	positive and negative reporter assay target validations https://www.ccb.uni-saarland.de/miratbase
	TissueAtlas	[47]	human, rat	miRNA	comprehensive atlas of miRNA expression in multiple organs https://www.ccb.uni-saarland.de/tissueatlas
	CellTypeAtlas	[48,49]	human	miRNA	comprehensive atlas of miRNA expression in multiple blood cell types https://www.ccb.uni-saarland.de/cf
	ATmiRes	[50]	human	miRNA	comprehensive atlas of miRNA expression in ancient samples https://www.ccb.uni-saarland.de/atmires
**Helper**	miRTaH	NA	all	miRNA	a tool for designing miRNA reporter assay experiments https://www.ccb.uni-saarland.de/mirtah
	miRBase Converter	[20,28]	all from miRBase	miRNA	converts miRNA identifiers between arbitrary miRBase versions https://www.ccb.uni-saarland.de/mieaa/mirna_version_converter
	miBlast	[7]	all from miRBase	miRNA	searches potential novel miRNAs in the miRCarta database https://www.ccb.uni-saarland.de/mircarta/miblast

### 3.7. Dependencies and Workflows

Dependencies and integration between the tools already exists. For example, if a miRTargetLink analysis has been done, the pathway enrichment can be directly instantiated from the results page, making use of the existing API. Another example is the information on target pathways of miRNA candidates from miRCarta. The most recent version of the pathway database notably also contains pre-computed target pathways for all candidates in miRCarta that are not contained in miRBase. Furthermore, if a miRMaster analysis has been completed, users can immediately carry out a miEAA enrichment analysis from the results tab showing the quantification of miRNAs. One last example is the TissueAtlas. For comparison, the user can select data to display from the ancient tissues directly besides the modern tissues by integrating AtmiRes. These represent limited efforts for a more integrated solution using APIs for the integration of the data. A list of potential associations that can be used to build comprehensive workflows is presented in Supplementary Materials Table S1. Here, for 33 combinations, the tools that can be used to build workflows are presented. Notably, other associations that might be reasonable and possible not included in this table exist.

**Figure 3:**
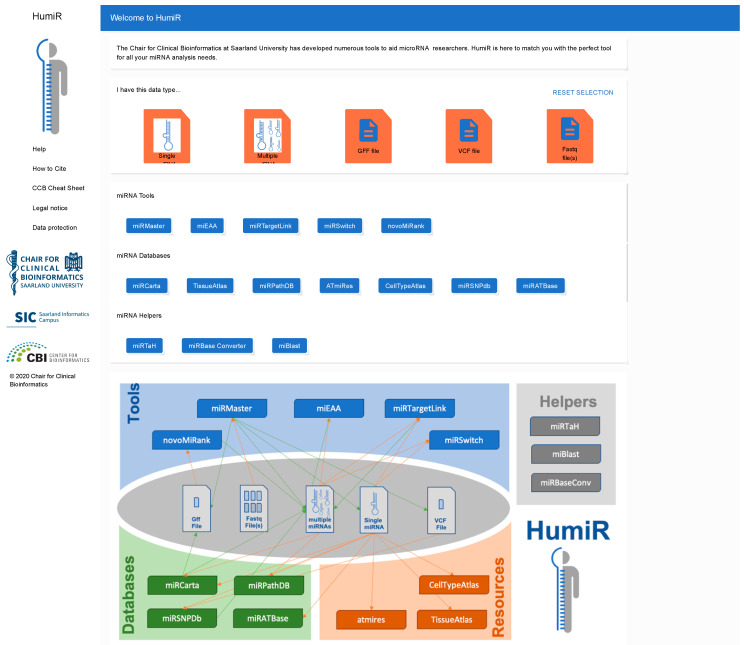
Web presence of HumiR. Screenshot of the web interface of HumiR. Users can select their input or output file types and all respective tools are enabled while all others are disabled. Although currently the workflows must be executed manually, the aim of HumiR is a fully integrated workflow management of all tools.

Let us evaluate a typical workflow. A user might have carried out a small RNA sequencing study on case and control samples. The first and straightforward step is to perform an analysis using miRMaster. As result the user gets a list of novel miRNAs. He can upload a respective gff file to NovoMiRank and exclude likely false positives. Next, he can search for the remaining candidates in miRCarta and knows whether they have been reported before. He also can directly extract targets and target pathways of those novel miRNAs. From the set of deregulated miRNAs, he can compute enriched or depleted functional categories directly from the miRMaster results page and having identified promising candidates, he can check which organs these are expressed in. This example workflow is presented in Figure 3. While all these single steps each take a few minutes, they require in total still a relevant amount of time. Further, many copy and paste or download actions are required. This currently limits taking full advantage of the different tools and databases. Nonetheless we provide the results of the analyses showing how much insights can be generated with minimal time. First, we executed miRMaster on the example data set of 70 Alzheimer’s cases and controls and downloaded all results files (117 files and 536 MB data). miRMaster computed 1605 expressed miRNAs from miRBase along with their p-values between Alzheimer’s disease (AD) and control. For the highly significant miRNAs (59 miRNAs; *p* < 0.005) we performed a standard miEAA analysis using all pathways and categories and downloaded the results. From the far over 10,000 categories, 901 remained significant in the analysis with the three most significant: “Alzheimers Disease Dysregulated” (*p* = 6.2 × 10^−27^), “Alzheimers Disease Downregulated” (*p* = 2.5 × 10^−17^) and “neuromyelitis optica” (*p* = 2.1 × 10^−14^). The next categories were a seed family (GCAAAG; *p* = 8.6 × 10^−8^) followed by the category “Alzheimers Disease” from MNDR (*p* = 3.3 × 10^−8^). This enrichment clearly validated that we indeed found relevant AD miRNAs in our study, most significantly hsa-miR-1468-5p; hsa-miR-144-5p; hsa-miR-3127-3p; hsa-miR-4781-3p; hsa-miR-6505-3p; hsa-miR-144-3p; hsa-miR-190a-5p; hsa-miR-26b-3p; hsa-miR-101-3p; hsa-miR-628-3p. Those we checked in the miRNA tissue atlas. While most miRNAs were found in the vein or in blood cells, several of those miRNAs were also expressed in the brain or related entities, e.g., miR-144-3p in the dura mater, hsa-miR-190a-5p in the brain and the dura mater, hsa-miR-26b-3p in the arachnoid mater, hsa-miR-101-3p in the brain, arachnoid mater and dura mater or hsa-miR-628-3p in the brain. Respective brain miRNAs might have a higher chance to be functionally related to the disease. A miRTargetLink analysis of the 59 miRNAs identified one core network consisting of 13 miRNAs, 38 target genes and 86 regulatory events between the miRNAs and target genes, among them APP, regulated by the brain miRNA hsa-miR-101-3p and hsa-miR-15a-5p. We next asked how many of the new miRNA candidates from this study have been previously reported and are stored in miRCarta. Of 8982 miRNA candidates reported in that study, 4661 (52%) are actually no new miRNA candidates but are already stored in miRCarta. Finally, we also checked which miRNAs perform arm shifts between case and control. To this end, the miRMaster output was directly transferred to miRSwitch. The analysis identified four miRNAs that vary in their arm usage between AD and control, three with dominant-3p arm (hsa-mir-17, hsa-mir-130b and hsa-mir-3200) and one with dominant -5p arm (hsa-let7d). Using a set of our tools we demonstrated how different tools can be integrated to achieve comprehensive results for a miRNA case-control sequencing study.

## 4. Outlook: The HumiR Knowledge Base

The previous analysis example demonstrated that a time efficient analysis of human small RNA sequencing data is well feasible with our tool collection. Few examples of direct and deep integration already demonstrate that the process can be further improved. The disadvantage still remains, we had to download and upload again many files between the different tools, calling for a fully integrated knowledge base and analysis resource of human small non-coding RNAs. With each update of a tool or database we now introduce a comprehensive RESTful API. This API describes the interfaces that are sketched in Figure 1 but are so flexible that they can be easily extended. Once available, HumiR will be updated by the integrated version. Our ambition is that users only provide their data. Reasonable workflows for the analysis of these data are automatically generated and proposed to the user. Before they are executed, they can be customized. All results of the different tools are then aggregated in one central place. This facilitates the simplest analyses (entering one single human miRNA and get all information related to this miRNA) up to very complex question such as “Which target pathways of a yet unknown miRNA are differentially expressed between cases and controls?” supported.

## Supplementary Materials

The following are available online at https://www.mdpi.com/2218-273X/10/11/1576/s1, Table S1: A list of potential associations that can be used to build comprehensive workflows.
