# Supplementary material for: HumiR: Web Services, Tools and Databases for Exploring Human microRNA Data
Source: Biomolecules. 2020 Nov 20;10(11):1576. doi: 10.3390/biom10111576 (PMC7699549; doi:10.3390/biom10111576)
Supplement: Supplementary file 1 [file biomolecules-10-01576-s001.pdf]

Tool 1	Tool 2
miRMaster	miEAA
miRMaster	miRTargetLink
miRMaster	miRSwitch
miRMaster	CellTypeAtlas
miRMaster	TissueAtals
miRMaster	ATmiRes
miRMaster	miRATbase
miRMaster	miRPathDB
miRMaster	miRSNPDb
miRMaster	miRCarta
miRMaster	NovoMiRank
NovoMiRank	miRCarta
NovoMiRank	miBlast
NovoMiRank	miRPathDB
NovoMiRank	miRTaH
miEAA	TissueAtals
miEAA	CellTypeAtlas
miEAA	atmiRes
miEAA	miRSwitch
miRTargetLin	TissueAtals
miRTargetLin	CellTypeAtlas
miRTargetLin	atmiRes
miRTargetLin	miRSwitch
CellTypeAtlas	miEAA
CellTypeAtlas	TissueAtlas
CellTypeAtlas	miRATbase
CellTypeAtlas	miRTaH
CellTypeAtlas	miRTargetLink
TissueAtlas	miEAA
TissueAtlas	TissueAtlas
TissueAtlas	miRATbase
TissueAtlas	miRTaH
TissueAtlas	miRTargetLink
